# Placental Transfusion and Cardiovascular Instability in the Preterm Infant

**DOI:** 10.3389/fped.2018.00039

**Published:** 2018-02-27

**Authors:** Zbynĕk Straňák, Simona Feyereislová, Peter Korček, Eugene Dempsey

**Affiliations:** ^1^Third Faculty of Medicine, Charles University, Prague, Czechia; ^2^Institute for the Care of Mother and Child, Prague, Czechia; ^3^Department of Paediatrics and Child Health, Neonatal Intensive Care Unit, University College Cork, Cork, Ireland; ^4^INFANT Centre, University College Cork, Cork, Ireland

**Keywords:** birth transition, placental transfusion, hypotension, neonatal outcome, very low birth weight

## Abstract

Postnatal adaptation in preterm newborn comprises complex physiological processes that involve significant changes in the circulatory and respiratory system. Increasing hemoglobin level and blood volume following placental transfusion may be of importance in enhancing arterial oxygen content, increasing cardiac output, and improving oxygen delivery. The European consensus on resuscitation of preterm infants recommends delayed cord clamping (DCC) for at least 60 s to promote placenta–fetal transfusion in uncompromised neonates. Recently, published meta-analyses suggest that DCC is associated with fewer infants requiring transfusions for anemia, a lower incidence of intraventricular hemorrhage, and lower risk for necrotizing enterocolitis. Umbilical cord milking (UCM) has the potential to avoid some disadvantages associated with DCC including the increased risk of hypothermia or delay in commencing manual ventilation. UCM represents an active form of blood transfer from placenta to neonate and may have some advantages over DCC. Moreover, both methods are associated with improvement in hemodynamic parameters and blood pressure within first hours after delivery compared to immediate cord clamping. Placental transfusion appears to be beneficial for the preterm uncompromised infant. Further studies are needed to evaluate simultaneous placental transfusion with resuscitation of deteriorating neonates. It would be of great interest for future research to investigate advantages of this approach further and to assess its impact on neonatal outcomes, particularly in extremely preterm infants.

## Introduction

Neonatal adaptation is a complex physiological process that involves numerous adaptive changes within several organ systems. In order to achieve this, an almost immediate transition to air breathing with intricate pressure changes as well as changes of blood flow in circulatory system must occur simultaneously, especially enhanced pulmonary blood flow. Adequate blood volume is necessary to facilitate these adaptive processes and ensure sufficient oxygen transport and organ/tissue perfusion. Increasing fetal hemoglobin (Hb) by placental transfusion may be considered a method in enhancing arterial oxygen content, increasing cardiac output, and improving oxygen delivery. It involves a shift of placental blood to the neonate within a limited amount of time immediately after delivery and may be achieved in two different ways: delayed cord clamping (DCC) and umbilical cord milking (UCM) (1–3).

These enhanced placental transfusion techniques can help to achieve a greater blood volume at birth (increase of up to 10–15 mL/kg), which may be significant especially in very low birth weight infants. Most of the blood volume (50–70%) is transferred within the first minute of life and can form up to 15% of overall blood volume of the neonate. Milking the cord four times provides a comparable amount of placental–fetal blood being transferred as performing DCC for at least 30 s (3, 4).

Recommendations with regards to the handling of umbilical cord have changed significantly over the last decades. The 2015 ILCOR and ERC guidelines advocate DCC (for at least 1 min) in uncompromised term and preterm infants. However, the authors advise that placental transfusion should be discontinued in infants who are not breathing, so that resuscitation measures are not delayed. The European consensus on resuscitation of the preterm infant recommends DCC (if possible) for at least 60 s to promote placental–fetal transfusion. Cord milking is considered a reasonable alternative if DCC is not possible (5–7).

The aim of this review is to present the recently published data with regards to different methods of placental transfusion, to compare and emphasize their impact in postnatal adaptation with particular emphasis on measures of cardiovascular stability in the first days of life in preterm infants.

## Rationale for Placental Transfusion

In optimal circumstances, appropriate aeration of the lungs, as achieved by spontaneous breathing, leads to increased pulmonary blood flow with a consequent increase in preload of the left ventricle (LV) and hence increased cerebral blood flow (8–10). However, after removing low resistance placental circulation by immediate cord clamping (ICC), there is a sudden increase in systemic arterial resistance of the neonate, which leads to increased afterload of the LV, as well as decreased preload of the right side of the heart (8–10). Furthermore, immaturity of myocardium in preterm infants means that they are vulnerable to rapid hemodynamic changes that can cause fluctuations in organ/tissue perfusion (3). In particular, the central nervous system appears to be most vulnerable to such changes, as cerebral autoregulation in preterm infants can be often impaired or absent in the immediate transitional period (11, 12).

In cases where cord clamping precedes onset of spontaneous ventilation, decreasing ventricular preload and ventricular output could potentially result in a decrease in cerebral blood flow. Low and altered cerebral blood flow has been associated with intraventricular hemorrhage (IVH). Thus, maintaining cerebral blood flow is an important component in reducing the incidence of severe IVH (8–10). In this respect, a published study using Near InfraRed Spectroscopy as well as several meta-analyses on this topic have shown that an adequate placental transfusion by DCC or UCM may increase cerebral blood flow during postnatal adaptation of the newborn and may help reduce IVH occurrence (10).

In addition, apart from lower rates of IVH, other benefits of enhanced placental transfusion in preterm neonates and newborns with extremely low birth weight have already been reported: higher Hb levels, reduced number of needed blood transfusions, lower risk for necrotizing enterocolitis, and higher mean blood pressure (BP) after birth (13, 14). Data relating to short-term hemodynamic variables are discussed in greater detail below.

## Placental Transfusion: Hemodynamic Effects

A number of studies highlight the potential cardiovascular benefits of placental transfusion in preterm infants. These include effects on mean BP, administration of volume and inotropes and other objective hemodynamic assessment methods [e.g., superior vena cava (SVC) blood flow, right ventricle output]. The studies are outlined in Table 1 and discussed in greater detail below (10, 12–21). We have grouped the studies as placental transfusion (either DCC or UCM) compared to ICC and then a comparison of alternative placental transfusion strategies, focusing primarily on markers of cardiovascular stability.

**Table 1:** Overview of randomized controlled trials regarding the methods of placental transfusion and their impact on hemodynamics in preterm infants.

Leading author (journal and year)	Reference	Placental transfusion	Number of patients	Gestational age	Hemodynamic background	Conclusions
Baenziger (Pediatrics 2007)	(10)	Delayed cord clamping (DCC) (60–90 s) vs immediate cord clamping (ICC)	39	30 (mean)	Cerebral blood volume was not different between two groups at the age of 4 and 24 h, and mean cerebral tissue oxygenation was higher in the DCC group at the age of 4 (4.4%) and 24 h (3.2%)	Delayed cord clamping improves cerebral oxygenation in preterm infants in the first 24 h. Mean arterial blood pressure (MABP) was higher in the DCC group at 4 h of age, but not at 24 h

Backes (Journal of Perinatology 2016)	(12)	DCC (30–45 s) vs ICC (<10 s)	40	22–27	Higher MABP in the first 24 h (4.13 mmHg difference) and significantly lower frequency of hypotension treatment were observed in the DCC group	DCC appears safe, feasible, and offers hematological and circulatory advantages
						No statistically significant differences were found in severe neonatal morbidities except for lower incidence of severe intraventricular hemorrhage (IVH) in the DCC group

Kugelman (American Journal of Perinatology 2007)	(13)	DCC (30–45 s) vs ICC (5–10 s)	65	<35	The DCC group tended to have higher initial diastolic blood pressure (BP) and higher hematocrit. Very low birth weight (VLBW) infants with DCC tended to have higher MABP and needed less mechanical ventilation and surfactant administration	DCC seems to be safe and may be beneficial (from ventilation and circulation point of view) when compared with ICC in premature infants

Sommers (Pediatrics 2012)	(14)	DCC vs ICC	51	<32 (24–31)	Higher superior vena cava flow (SVCF) over the study period and greater right ventricular output (RVO) at 48 h of life in the DCC group were observed. No difference in other parameters (middle cerebral artery velocity, left ventricle shortening fraction, patent ductus arteriosus (PDA), MABP) were described	DCC in preterm infants is associated with potentially beneficial hemodynamic changes over the first days of life

Katheria (Journal of Pediatrics 2014)	(15)	Umbilical cord milking (UCM) vs ICC	60	<32	Systemic blood flow (SVCF, RVO) in the first 6 and 30 h of life was higher in the UCM group	Greater systemic blood flow was demonstrated with UCM in preterm neonates
						The UCM group also had fewer days on oxygen therapy and less frequent use of oxygen at 36 weeks of corrected postmenstrual age

Ibrahim (Journal of Perinatology 2000)	(16)	DCC (20 s) vs ICC	32	24–28	Higher MABP at 4 h of life and lower need of albumin transfusion to stabilize blood pressure and increase tissue perfusion in the first 24 h were recorded in the DCC group	DCC significantly reduced the requirement for albumin transfusion. DCC also increased the initial hematocrit, hemoglobin levels, and MABP. The risks for PDA/IVH were similar in both groups

Katheria (Pediatrics 2015)	(17)	DCC (45–60 s) vs UCM	197	<32 (23–31)	Systemic blood flow (SVCF, RVO) in the first 12 h was higher in the UCM group. MABP over the first 15 h and urine output in the first 24 h were higher in the UCM group	UCM provides greater placental transfusion when compared to DCC, especially in preterm infants born by cesarean delivery. Risk for any IVH was lower in the UCM group. Other neonatal morbidities were similar

March (Journal of Perinatology 2013)	(18)	UCM vs ICC	75	24–28	Initial systolic and diastolic BPs were higher in the UCM group (difference of 2.5 mmHg for systolic and 1 mmHg for diastolic pressure). Differences were not statistically significant	Infants in the UCM group were significantly less likely to develop any IVH (incidence of 25% in the UCM group vs 51% in the ICC group). However, the incidence of severe IVH was similar in both groups

Oh (Journal of Perinatology 2011)	(19)	DCC (30–45 s) vs ICC (<10 s)	33	24–28	No difference observed between groups in hourly MABP in the first 12 h of life	DCC offers effective placento–fetal transfusion in VLBW infants—trend toward higher hematocrit in the first 6 weeks of life. However, no statistically significant differences in neonatal morbidities between groups were demonstrated

Mercer (Journal of Pediatrics 2016)	(20)	DCC (30–45 s) vs ICC (<10 s)	202	<32	No difference in the admission MABP between groups	There were no differences in rates of IVH; however, the DCC group had better motor performance at 18–22 months of corrected age (Bayley Scales of Infant Development third Edition)

Popat (Journal of Pediatrics 2016)	(21)	DCC (≥60 s) vs ICC (<10 s)	266	<30	No difference between groups in SVCF measurement; however, the DCC group had lower RVO. Rates of treated hypotension, PDA size, and its treatment were similar	DCC had no effect on systemic blood flow in preterm infants measured as SVCF in the first 24 h

## Comparison 1: DCC Compared to ICC

A previous meta-analysis of 15 studies examined 738 infants born before 37 weeks of gestation, having either DCC or UCM compared to ICC and found no significant differences in a number of outcome variables including Apgar scores, extent of resuscitation, incidence of hypothermia, polycythemia, and various pulmonary outcomes (ventilatory support, oxygen dependence). However, the following benefits were noted: DCC was associated with fewer infants requiring blood transfusions [risk ratio (RR) 0.61, 95% confidence interval (CI) 0.46–0.81], less IVH (ultrasound diagnosis of all grades: RR 0.59, 95% CI = 0.41–0.85), lower risk for necrotizing enterocolitis (RR 0.62, 95% CI = 0.43–0.90), and a lesser need for inotropic support (RR 0.42, 95% CI = 0.23–0.77) (22). However, there were only four studies reporting incidence and treatment of hypotension. The mean arterial blood pressure (MABP) was significantly higher in those allocated to DCC. Two studies (13, 23) present data at birth (97 infants, MD 3.52, 95% CI 0.60–6.45) and two other studies (10, 24) at age 4 h (111 infants, MD 2.49, 95% CI 0.26–4.72).

A number of more recent studies comparing DCC with ICC have reported BP data. Backes et al. found that infants who received DCC, as compared to those with ICC, had a higher BP within the first 24 h of life (mean difference of 4.13 mmHg, 95% CI 2.0 to 6.2, *P* < 0.01) and infants in the ICC group received significantly more treatment for hypotension (ICC 45% vs DCC 12%, *P* < 0.01). Interestingly, there was a trend toward lower rates of severe IVH, particularly for the most preterm infants (22–24 weeks of gestation) (12).

Another study addressing various hemodynamic outcomes included over 200 patients. There was no significant difference between groups in the mean BP values and the lowest SVC flow [ICC 71.4 mL/kg/min (SD 28.1) vs DCC 70.2 mL/kg/min (SD 26.9); *P* = 0.7]. The group with DCC also had lower right ventricular output (RVO) (−21.9 mL/kg/min, 95% CI −39.0, −4.7; *P* = 0.01). Rates of interventions for hypotension did not differ between the groups (21).

Since the abovementioned reviews, the largest randomized trial of DCC compared to ICC has been reported by Australian Placental Transfusion Study Collaborative group (25). The study revealed no significant difference in the primary outcome (death or major morbidity at 36 weeks of gestation) between infants assigned to DCC (37.0%) and those assigned to ICC (37.2%) group (RR 1.00; 95% CI, 0.88–1.13; *P* = 0.96). The mortality was 6.4% in the DCC group, and 9.0% in the ICC group (*P* = 0.03 in unadjusted analyses; *P* = 0.39 after *post hoc* adjustment for multiple secondary outcomes). Limitation of this study was that a considerable percentage (27%) of infants who were randomized to DCC group received ICC instead. The major reason for this was concerns about the infants well being (25). Moreover, similar limitations have been found in another large study, where the reasons included, in addition, implementation issues including miscommunication, concern about the mother, and fetal concern (21).

## Comparison 2: UCM Compared to ICC

Umbilical cord milking has the potential to avoid some perceived disadvantages of DCC such as potential delay in life saving therapy of the sick neonate and the risk of hypothermia (2). However, the exact description of UCM technique varies between published studies, in particular, in terms of the number of times the stripping of umbilical cord is performed, the definition of the milking speed, and in whether the umbilical cord is cut before or after the cord milking has been performed. The milking speed of 20 cm/2s and the frequency of cord stripping of 3–5 times are most commonly used in published cases.

A recent meta-analysis of 7 clinical trials involving 501 neonates compared UCM to ICC. Five studies included 277 infants born before <33 weeks. This review found that milking the cord was associated with higher Hb levels (mean difference, 20 g/L, 95% CI = 13–27), lower risk of IVH of all grades (RR 0.62, 95% CI = 0.41–0.93), and lower oxygen requirement at 36 weeks postmenstrual age (RR 0.42, 95% CI = 0.21–0.83). There was no statistically significant difference in mortality. When specifically addressing hypotension and its treatment, the authors found no difference in the incidence of hypotension in the first 24 h requiring treatment with volume expanders or support with inotropes between the two groups (11).

Katheria and colleagues randomized 60 neonates to UCM or ICC. They showed improved immediate transition in the delivery room. Neonates randomized to cord milking had a significantly higher mean and diastolic BP at 6 h. Infants in the milking arm had higher SVC flow in the first 6 h (98 ± 27 vs 66 ± 18 mL/kg/min, *P* < 0.001), greater hematocrite at 12 h (46 ± 10 vs 38 ± 7 mg/dL, *P* = 0.02), and were less likely to receive a blood transfusion (9/14 vs 14/14 infants, *P* = 0.04) compared with infants with ICC (15).

Hosono et al. present data on 40 preterm infants who were randomized to UCM or ICC. UCM was associated with higher initial BP values. The initial systolic, diastolic, and mean BPs in the milked group were significantly higher than in the control group (*P* < 0.05). This translated into a greater proportion of infants receiving inotropes in the control arm (26).

Conversely, March et al. found no difference between early systolic pressure values [3.0 (37.0–51.0) vs 40.5 (36.0–46.5), *P*-value 0.32] and diastolic BP values [22.0 (18.0–29.0) vs 21.0 (15.0–28.5) *P*-value 0.68] (18). In what appears to be a pre/post type study, Takami et al. report improved MABP on admission (mmHg) 28 (3.2) vs 35 (4.3) *P*-value <0.05, an improved urine output within 24 h (mL/kg) 1.1 (1.1) vs 1.9 (1.3) *P*-value <0.05, and less need for volume expansion 58 vs 19% *P*-value <0.05 (27). They also recorded measures of cardiac output and cerebral oxygenation. Both LVCO and SVC flow in the milked group were significantly higher than those in the control group. The TOI in the milked group was significantly higher than in the control group over the first 36 h after birth. However, these findings need to be interpreted in light of potential biases in trial design.

## Comparison 3: DCC and UCM

The key methodological differences between DCC and UCM have been highlighted previously. DCC or UCM may not be of equal benefit for all preterm newborns. In neonates born by cesarean delivery, more blood can potentially remain in the placenta at birth due to anesthetic and surgical interventions that interfere with active uterine contractions.

In a two-center trial involving 197 neonates born at less than 32 weeks of gestation, Katheria et al. demonstrated that UCM seems to be a preferential method of placental transfusion in neonates born by cesarean delivery (17). The UCM (4 strippings) group had higher SVC blood flow and RVO measures in the first 12 h of life, a higher Hb level, better body temperature in delivery room, higher BP in the first 15 h, and higher urine output in the first 24 h of life, as compared to the DCC (45–60 s) group. There were no differences between the DCC and UCM groups for neonates born vaginally. There were no differences in cerebral oxygen saturation or cardiac output as measured by impedance (17).

Rabe et al. also compared UCM (4 strippings) and DCC (30 s) in a similar population group (neonates of less than 33 weeks of gestation at birth), but found no difference between the groups in both the admission Hb levels or the number of blood transfusions required in the first 6 weeks of life. This trial, however, comprised of only 58 randomized neonates (31 in DCC and 27 in UCM group), and the mode of delivery was not accounted for (28).

## Comparison 4: Ventilation with DCC Compared to DCC Alone

One trial has compared DCC alone to ventilation with DCC. In this trial, Katheria et al. compared 62 infants with DCC alone to 63 infants with ventilation with DCC and found no differences between a large number of early hemodynamic changes (cerebral oxygenation by near-infrared spectroscopy, cardiac output and stroke volume by electrical cardiometry, or SVC flow by functional echocardiography) (29).

## Summary of Haemodynamic Effects

Recent data demonstrate that DCC or UCM is advantageous over ICC. Despite a number of trials performed to date, the evidence for improved cardiovascular adaptation is somewhat inconsistent. What is clear is that placental transfusion, either as DCC or UCM, compared to ICC is not associated with an adverse effect on BP, the need for volume administration or inotrope use. The majority of studies that report on these outcomes either show no difference or else an improvement in the placental transfusion arm.

Very few trials report on measures of cardiac output and the largest study that presented data on SVC flow and RVO had a high crossover rate from DCC to ICC. This trial found no difference in SVC flow and a reduction in RVO in the ICC group (21). Conversely, milking was associated with an improvement in both SVC flow and RVO, suggesting that UCM may be a better alternative to DCC in cardiovascular adaptation. These questions remain to be answered by future studies.

## Cardiovascular Adaptation and Long-Term Outcomes

The potential benefit of placental transfusion with regards to neurodevelopmental outcome may be multifactorial. Enhanced cardiovascular adaptation resulting in improved cardiac output and improved cerebral blood flow may play an important role. The potential benefits may result in a reduction in brain injury as evidenced by reduced rates of IVH. In addition to these potential changes, improved blood volume resulting in increased iron content may also be neuroprotective. Furthermore, as cord blood is known to contain hematopoietic stem cells at birth, neonates may possibly benefit from their neuroprotective or even neurorestorative effect (20). Mercer et al. reported on preterm infants of less than 32 weeks of gestation, who received either ICC (in less than 10 s) or DCC (after 30–45 s). While there was no difference in the first recorded mean BP, rates of IVH or late onset sepsis between the groups, the DCC group showed better motor performance at 18–22 months corrected age (Bayley Scales of Infant Development, Third Edition) suggesting other potential mechanisms (20). Long-term follow-up of completed trials and future studies will help to clarify such potential benefits.

A recently published study of 197 infants showed those randomized to UCM had higher language and cognitive scores compared with those randomized to DCC. There was no difference in rates of mild or moderate to severe neurodevelopmental impairment (17).

## Conclusion

Many questions remain unanswered in relation to placental transfusion and its potential hemodynamic effects. UCM may have advantages over DCC by reducing the incidence of morbidities, the number of needed blood transfusions, and the requirement for supplemental oxygen at 36 weeks postmenstrual age. UCM may be of greater benefit than DCC to neonates born by cesarean section and a significant proportion of preterm neonates tend to be delivered by this method. UCM is more likely to be readily complied with than DCC due to fewer concerns of delaying commencement of care for the preterm neonate, as was evidenced in the recent RCT where 27% of patients assigned to DCC had ICC performed instead for this reason (25).

Reassuringly a recent meta-analysis by Backes et al. show that there seem to be no differences regarding safety measures observed (5-min Apgar scores, admission temperatures of neonates, incidence of delivery room intubation, peak serum bilirubin levels) when using DCC or UCM (30).

Many of the published clinical studies regarding placental transfusion have excluded neonates with the need for resuscitation at delivery, limiting the generalizability of the findings. Studies that include these patients are hence needed. There are, however, a number of planned and ongoing studies in this area. Some of these studies include short term markers of cardiocerebral adaptation incorporating echocardiography measures and near infrared spectroscopy. One such trial is the PREMOD 2 study (NCT 01866982), which includes detailed assessment of preterm infants of less than 28 weeks gestation in the delivery room and during the first 24 h of life. Infants will receive DCC or UCM and will have measures of cerebral oxygenation and echocardiography performed, which will hopefully provide further insight into the (patho-)physiological changes that are occurring over this time. Additional studies regarding neurodevelopmental outcomes, specifically in preterm neonates having had DCC in comparison to UCM performed would also be of great importance.

## Author Contributions

ZS, SF, and PK: these authors contributed equally to this work—substantially contributed to the conception of the work, drafted and reviewed the article. SF and PK: analysis and interpretation of presented data. ED: reviewed the article for important intellectual content. All authors gave final approval of the version to be published and agreed to be accountable for all aspects of the work.

## Conflict of Interest Statement

The authors state that there are no conflicts of interest regarding the publication of the article.

## Abbreviations

BP, blood pressure; CI, confidence interval; DCC, delayed cord clamping; ELBW, extremely low birth weight; GA, gestational age; ICC, immediate cord clamping; IVH, intraventricular hemorrhage; LV, left ventricle; MABP, mean arterial blood pressure; MCA, middle cerebral artery; PDA, patent ductus arteriosus; SVC, superior vena cava; SVCF, superior vena cava flow; RR, risk ratio; RVO, right ventricular output; UCM, umbilical cord milking; VLBW, very low birth weight.
